# 342. Evaluation of Patient Satisfaction with Outpatient Parenteral Antibiotic Therapy (OPAT) at a University Medical Center

**DOI:** 10.1093/ofid/ofad500.413

**Published:** 2023-11-27

**Authors:** Ciara Sawey, Sapan A Munsiff, Colleen Burgoyne

**Affiliations:** University of Rochester, San Carlos, California; New York University, Pittsford, New York; Univ. of Rochester, Rochester, New York

## Abstract

**Background:**

With the expansion of OPAT care over recent years, new devices such as elastomeric balls have been added to the available drug delivery options, which also include syringe, electronic pumps and gravity bags. The primary objective of this study was to evaluate the experience of patients (and their caregivers) on OPAT, ease of use with various delivery methods, and barriers to outpatient care.

**Methods:**

The survey included questions on IV antibiotic received, delivery method, who administered the medicines, effect on daily activities, ease of access to staff, and thoughts on various aspects of the process and experience. Questions were multiple choice, based on Likert scale, or open-ended. Patients were recruited in person during infectious diseases (ID) clinic visits, or by phone, just before or around the time OPAT ended.

**Results:**

We had 51 responses from 130 patients from 10/1/22 thru 3/31/23. Median age group was 55-64 years, 45% were women, for 78% it was first OPAT episode, and 23% received > one IV drug. Syringe push was used for 37%, elastomeric ball for 38%, gravity bag for 18% and electronic pump for 7% of agents. Most (76%) received help in administering treatment by friend or family (spouse=75%). 44% of infusions were once/day and 27% were 3 or more times/day. Home nursing services were received by 94% (5 different agencies) and > 6 different infusion pharmacies supplied the medicines. Medicine was always delivered on time for 38 of 45 respondents.

All patients found IV push and elastomeric ball easy to use; 25% disagreed with this about gravity bag or electronic pump. Overall satisfaction was 94% with OPAT course and 94% felt instructions on how to administer medicines at home were clear and easy. Financial hardships were noted by 9 of 48 respondents (12 responded “neutral”) and 77% had to curtail some or most of their daily activities while on OPAT.

**Conclusion:**

Overall, patients’ responses were positive of our OPAT program. However, the large portion of patients needing to curtail their usual activities highlights the need for more study on how regimen details such as delivery device and infusion frequency impact patient and caregiver experience. Financial hardships may be underestimated as final bills may not yet have been received by patients.

**Disclosures:**

**All Authors**: No reported disclosures

